# A risk model and nomogram for high-frequency hearing loss in noise-exposed workers

**DOI:** 10.1186/s12889-021-10730-y

**Published:** 2021-04-17

**Authors:** Ruican Sun, Weiwei Shang, Yingqiong Cao, Yajia Lan

**Affiliations:** 1 Department of Occupational and Environmental Health, West China School of Public Health and West China Fourth Hospital, Sichuan University, Chengdu, Sichuan China; 2 Department of Occupational Health and Radial Control, Sichuan Center for Disease Control and Prevention, Chengdu, Sichuan China; 3 Department of Occupational Disease Prevention and Control, Pidu District Center for Disease Control and Prevention, Chengdu, Sichuan China

**Keywords:** High-frequency hearing loss, Risk model, Nomogram

## Abstract

**Background:**

High-frequency hearing loss is a significant occupational health concern in many countries, and early identification can be effective for preventing hearing loss. The study aims to construct and validate a risk model for HFHL, and develop a nomogram for predicting the individual risk in noise-exposed workers.

**Methods:**

The current research used archival data from the National Key Occupational Diseases Survey-Sichuan conducted in China from 2014 to 2017. A total of 32,121 noise-exposed workers completed the survey, of whom 80% workers (*n* = 25,732) comprised the training cohort for risk model development and 20% workers (*n* = 6389) constituted the validation cohort for model validation. The risk model and nomogram were constructed using binary logistic models. The effectiveness and calibration of the model were evaluated with the receiver operating characteristic curve and calibration plots, respectively.

**Results:**

A total of 10.06% of noise-exposed workers had HFHL. Age (OR = 1.09, 95% CI: 1.083–1.104), male sex (OR = 3.25, 95% CI: 2.85–3.702), noise exposure duration (NED) (OR = 1.15, 95% CI: 1.093–1.201), and a history of working in manufacturing (OR = 1.50, 95% CI: 1.314–1.713), construction (OR = 2.29, 95% CI: 1.531–3.421), mining (OR = 2.63, 95% CI: 2.238–3.081), or for a private-owned enterprise (POE) (OR = 1.33, 95% CI: 1.202–1.476) were associated with an increased risk of HFHL (*P* < 0.05).

**Conclusions:**

The risk model and nomogram for HFHL can be used in application-oriented research on the prevention and management of HFHL in workplaces with high levels of noise exposure.

**Supplementary Information:**

The online version contains supplementary material available at 10.1186/s12889-021-10730-y.

## Introduction

Occupational hearing loss is the second most common form of sensory-related hearing loss after age-related hearing loss and is a major occupation-related condition worldwide [1]. The World Health Organization (WHO) reported that 16% of adult hearing loss cases were caused by occupational noise exposure [2]. The WHO also indicated that the number of individuals with hearing loss could increase to 630 million by 2030 and may reach more than 900 million by 2050 [3].

As one of the largest global producers, China has many manufacturing, construction, and mining enterprises [4]. Noise pollution has become one of the key public hazards [5]. Due to the high cost of occupational noise services, mismanagement of occupational health and insufficient personal protective equipment for noise-exposed workers [6], the National Health and Family Planning Commission (NHFPC) of the People’s Republic of China (PRC) reported that the incidence of occupational ear, nose and throat diseases (OENTD) has increased in recent years. New cases of OENTD exceeded those of occupational poisoning to become the second most common occupational disease after occupational pneumoconiosis since 2015, and 95.90% of OENTD cases were noise-induced hearing loss (NIHL). From 2015 to 2019, the NHFPC reported that the numbers of new cases of OENTD were 1097 cases, 1276 cases, 1608 cases, 1528 cases, and 1623 cases, respectively [7–11].

In 2009, the China Centers for Disease Control and Prevention (CDC) launched the National Key Occupational Diseases Survey (NKODS), a nationwide surveillance project of ten key occupational hazards, namely, coal dust (coal silica dust), silica dust, asbestos, benzene, lead, noise, brucella, welding fumes, carbon disulfide, and phosphorus compounds, to cope with the increasingly severe challenges of occupational diseases. Noise is one of the ten key occupational hazards in the NKODS. The NKODS-Sichuan is the provincial level surveillance project of the NKODS.

HFHL is a characteristic of occupational hearing loss that develops slowly, affecting the higher frequencies first and extending gradually to the lower frequencies [12]. Previous studies focused on the prevalence or factors of HFHL in different regions [13–17], HFHL-related diseases [18–20], and HFHL-related mental disorders [21–23]. Regarding the risk model, the researchers paid more attention to the modelling of noise exposure risk in the workplace. However, few studies have provided risk models of HFHL or hearing loss. Lewkowski et al. [24] identified the risk of miners for developing occupational hearing loss and they found that miners who used planers, sanders, grinders, large machinery, and power hammers in work tended to suffer a high-level noise exposure. Pentti Kuronen et al. [25] constructed a risk model of NIHL in military pilots. It is well known that pilots have better health surveillance and management than other professions. Hong et al. [26] developed a prediction model using 379 South African adults, the predictors included the CD4 count, age, serum albumin level, and body mass index. This model can identify participants with drug resistant tuberculosis who are at high risk of developing minoglycoside-induced hearing loss. This research modelling the risk of disease-related hearing loss is not the same as the hearing loss induced by occupational noise. Therefore, this risk model is not appropriate for predicting HFHL in noise-exposed workers. Kuang et al. [27] predicted the individual risk of HFHL in 822 machinists. The predictors included age, sex male, limited earplug wearing, and high noise intensity. These results provide theoretical support for the prevention of HFHL among machinists. However, this study still has some limitations. On the one hand, the study was limited by its single-centre design, small sample size, and industry type whereby all the objects were mechanists. On the other hand, the study lacked an additional set of objects for external validation as well as a cut-off point to identify the high and low risk groups of workers. Furthermore, most studies have shown that the industry type and enterprise type are related to hearing loss [28]. The early prediction and diagnosis of HFHL in different industrial and enterprise types could provide evidence for the comprehensive prevention of HFHL.

Nomograms are pictorial representations of a complex mathematical formula and are reliable and convenient statistical predictive tools based on the indicators of a clinical event, enabling the calculation of an individual probability with a simple and visual graphical representation [29]. Nomograms are widely used to predict the probability of the higher risk of disease in clinical, which is usually used to predict survival in cancer patients, especially prostate cancer and breast cancer [30, 31]. Few studies on the development of a nomogram for HFHL have been published. As workers in high noise environments are at a high risk of hearing loss, especially HFHL, a risk model and nomogram may be valuable for management and prevention in workplaces with high levels of noise exposure. The approaches for assessing occupational noise exposure have been tested, such as questionnaire-based algorithms [32] and artificial intelligence [33]. However, those studies assessed noise exposure in the workplace or risk assessment of different task. Based on the current situation of HFHL, the development of a predictive tool using the NKODS dataset is a valuable effort for applied research on HFHL prevention. Besides, Chinese researchers have suggested that a predictive tool based on the NKODS dataset would be valuable in China to help regulators and managers effectively use the NKODS dataset and provide early warning for populations at risk for HFHL [34].

Considering the above-mentioned information, the objective of this study was to construct a risk model to identify the predictors of HFHL and develop a HFHL nomogram to calculate the individual risk for noise-exposed workers. The results of this study are expected to provide technical support and enhance application-oriented research on HFHL.

## Materials and methods

### Research data collection

In the NKODS-Sichuan, every trained health technicians collected the data of basic information and audiometric testing for noise-exposed workers, and entered the data into the NKODS-Sichuan Report System. In this study, we used the basic information and audiometric testing data from the NKODS-Sichuan dataset, collected from 2014 to 2017. The basic information was comprised of two sections: (1) company information including the company name, address, industry type, and enterprise type; and (2) personal information including the worker’s name, phone number, sex, date of birth, NED, exposure to occupational hazards, medical history and family history.

The audiometric testing consisted of a pure tone audiogram (PTA) examination. According to the national standard, Technical Specifications for Occupational Health Surveillance (GBZ 188–2014), audiometric testing were preceded by a period of at least 48 h without exposure to occupational noise. A noisy environment was defined as one with a noise equivalent intensity level greater than 80 dB based on the national Guidelines for Risk Management of Occupational Noise Hazard (AQ/T 4276-2016). The testing was carried out in a sound-isolating room [background noise less than 30 dB] in designated hospitals by trained health technicians. The audiometric testing consisted of: (1) Routine examinations of both ears. The routine examinations including the otoscopic examination was performed for each worker by an otolaryngologist to detect any ear pathology potentially affecting hearing function. The examination checklist also included eustachian tube function test, vestibular function test. (2) Thresholds were obtained three times at six different frequencies (0.5, 1, 2, 3, 4 and 6 kHz), with an interval between tests of at least 3 days. The BHFTA was used to diagnose HFHL, which was calculated using the arithmetic mean of the hearing thresholds at 3, 4, and 6 kHz in both ears. According to the national guidelines of Technical Specifications for Occupational Health Surveillance(GBZ 188–2014), 8 kHz was not included in the audiometric testing. The diagnostic gradation for NIHL was calculated based on the minimum threshold of three consecutive audiometric tests for each frequency (PTA according to the Chinese guidelines GB/T 7583 and GB/T 16403). In our study, PTA mainly consisted of air conduction testing for each worker in the NKODS. If noise-exposed workers had abnormal hearing levels (BHFTA≥40 dB), bone conduction measurements were used to determine whether occupational hearing loss had occurred. To exclude age-related hearing loss, hearing threshold levels were adjusted for age. According to the national standard, GB/T 7582–2004, the values for different age groups and frequencies, as shown in the Supplementary material Table 1, were subtracted from the measured hearing threshold value, yielding the age-adjusted hearing threshold value.

HFHL level categories were determined. Based on the Chinese national standard, Diagnosis of Occupational NIHL (GBZ 49–2014), and national guidelines, Risk Management of Occupational Noise Hazard (AQ/T 4276–2016), HFHL was defined as a BHFTA greater than or equal to 40 dB at 3,4, and 6 kHz. The categories of HFHL are shown in Table 1.

**Table 1:** Categories of HFHL levels

HFHL level	BHFTA (dB)
Normal hearing	≤25
Suspected HFHL	26–39
HFHL	40–79
Severe HFHL	≥80

*Abbreviations*: *HFHL* high-frequency hearing loss, *BHFTA* binaural high-frequency threshold average

Footnote. According to the General Guidelines for the Diagnosis of Occupational Diseases (GBZ/T 265–2014), HFHL is defined as a BHFTA ≥40 dB

### Research subjects

In this study, the inclusion criteria were as follows:

- the subjects had complete NKODS data and health examination reports from 2014 to 2017;
- the subjects were continuously exposed to noise in the workplace and were only exposed to noise;
- the NED was > 1 year;
- the subjects were older than 18 years old and younger than 50 years old.

The exclusion criteria were as follows:

- a family history of ear trauma or middle/external ear disease;
- a history of toxic drug and chemical exposure (such as organic solvents and carbon monoxide).

**Fig. 1:**
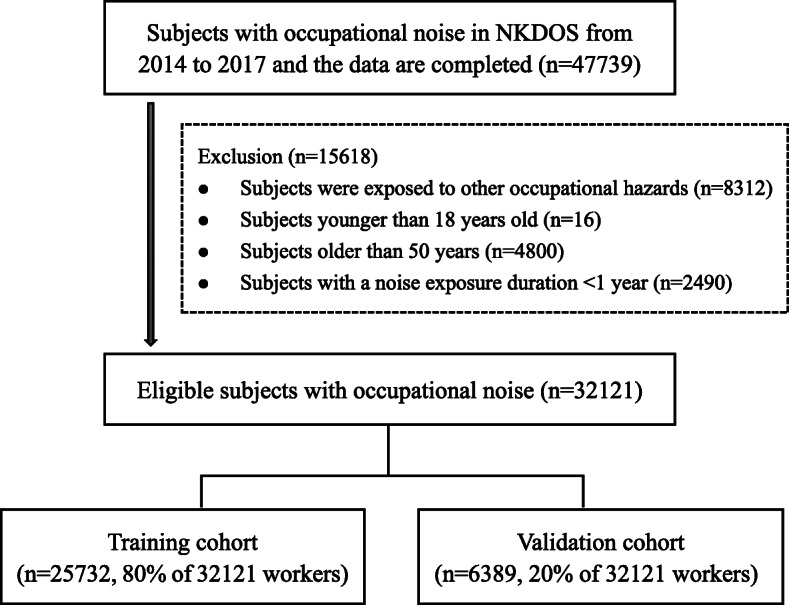
Study flowchart. *Abbreviation.* NKODS = national key occupational disease survey

A total of 47,739 subjects were exposed to occupational noise in Sichuan, China. According to the inclusion and exclusion criteria, 32,121 eligible subjects were enrolled in the study. All subjects were randomly divided into five datasets; 80% of the subjects were included as the training cohort, and 20% were included as the external validation cohort. The study flowchart is shown in Fig. 1.

### Data analysis

The characteristics of the training and validation cohorts are described by the frequency and percentage. Categorical variables are expressed as frequencies (%) and were compared by chi-square tests among all subjects.

Binary logistic regression analyses were used to identify the independent predictors of HFHL among the variables of sex, age, NED, industry type and enterprise type. HFHL was processed as a binary outcome in the model, where “0” indicated a BHFTA < 40 dB and “1” indicated a BHFTA ≥40 dB. The risks are expressed as odds ratios (ORs) and 95% confidence intervals (CIs).

A nomogram was established based on the risk model. The receiver operating characteristic (ROC) curve was used to examine the effectiveness of the risk model. The C-index was expressed by the area under the curve (AUC), which reflected the ability of the model to discriminate between those who would suffer HFHL from those who would not. Calibration plots were used to assess the nomogram’s calibration, which refers to how close the risk predicted by the nomogram is to the risk actually observed. To ensure the applicability of the nomogram in clinical practice, the cut-off value corresponding to the maximum Youden’s index was used to stratify HFHL workers into high and low risk groups. A two-tailed *P*-value< 0.05 was considered statistically significant. All statistical calculations were performed using *R* statistical software (version 3.5.0).

## Results

### Subject characteristics and the prevalence of HFHL

Of the 32,121 workers in the study, 73.66% (*n* = 23,661) were male and 26.34% (*n* = 8460) were female. The average age of the subjects was 38.41 years (SD = 7.98 years). Workers exposed to noise had a mean exposure duration of 8.63 years (SD = 7.86 years). The demographic and occupational characteristics of the subjects are summarized in Table 2.

**Table 2:** Characteristics of noise-exposed workers

Variable	All subjects		Training cohort		Validation cohort	
	(*n =* 32,121)		(*n* = 25,732)		(***n =*** 6389)	
	n	*%*	n	*%*	n	*%*
Sex						
Male	23,661	73.66	6828	73.46	1632	74.46
Female	8460	26.34	18,904	26.54	4757	25.54
Age (years)						
< 25	1908	5.94	1525	5.93	383	5.99
25–29	4191	13.05	3369	13.09	822	12.87
30–34	3888	12.10	3120	12.12	768	12.02
35–39	4559	14.19	3675	14.28	884	13.84
40–44	8868	27.61	7085	27.53	1783	27.9
≥ 45	8707	27.11	6958	27.04	1749	27.38
NED (years)						
0–4	13,511	42.06	10,840	42.13	2671	41.81
5–9	8137	25.33	6521	25.34	1616	25.29
10–14	3742	11.65	2967	11.53	775	12.13
15–19	2006	6.25	1625	6.32	381	5.96
20–24	2775	8.64	2227	8.65	548	8.58
25–29	1441	4.49	1152	4.48	289	4.52
≥ 30	509	1.58	400	1.55	109	1.71
Industry type						
Manufacturing	23,295	72.52	18,673	72.57	4622	72.34
Construction	294	0.92	232	0.90	62	0.97
Mining	3341	10.40	2670	10.38	671	10.51
Others ^a^	5191	16.16	4157	16.15	1034	16.18
Enterprise type						
SOE	9661	30.08	7762	30.16	1899	29.72
FOE	2446	7.61	1978	7.69	468	7.33
POE	20,014	62.31	15,992	62.15	4022	62.95

*Abbreviations*: *NED* noise exposure duration, *FOE* foreign-owned enterprise, *SOE* state-owned enterprise, *POE* private-owned enterprise

^a^Others included the transportation industry, storage industry, postal industry, agricultural industry, and fishery and animal husbandry industry

**Fig. 2:**
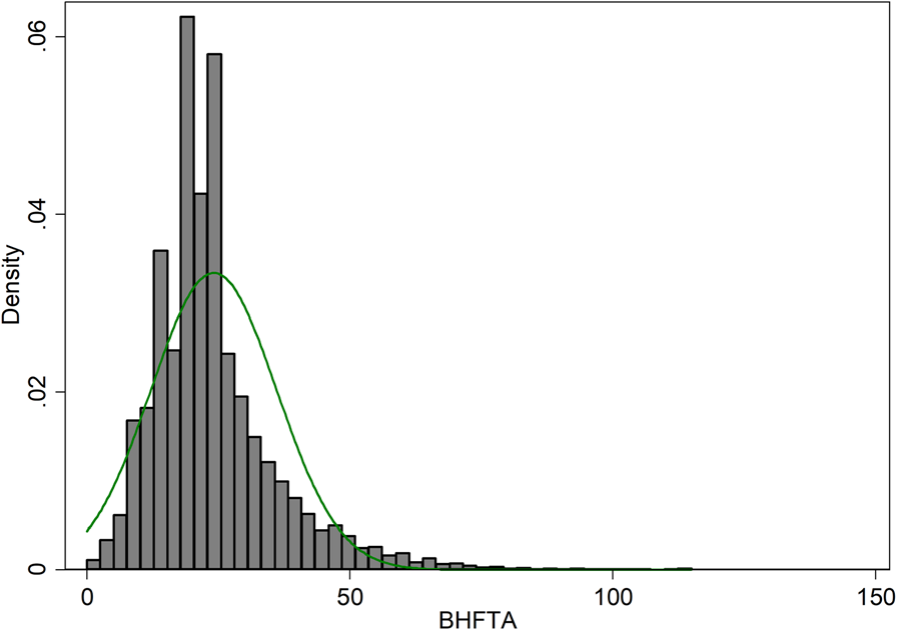
The distribution of hearing levels among all subjects (*n =* 23,121)

Among the 32,121 noise-exposed workers in this study, the lowest BHFTA was 0 dB, and the highest was 115 dB. Concerning the prevalence of hearing levels, 62.12% (*n* = 19,952) of the workers had a normal hearing level (BHFTA≤25 dB), 27.82% (*n* = 8937) of the workers had a BHFTA 26–39 dB, 9.78% (*n* = 3141) of the workers had a BHFTA 40–79 dB, and 0.28% (*n* = 91) of the workers had a BHFTA≥80 dB. The distribution of the BHFTA was expressed according to the approximately normal distribution of all parameters (Fig. 2).

### Different characteristics of the BHFTA in all subjects

Table 3 shows that the prevalence of HFHL increased stepwise according to age and NED (*P* < 0.05). Workers employed in manufacturing industries and mining industries were more likely to have HFHL than workers employed in construction and other industries (*P* < 0.05). In addition, those in POE had a significantly higher BHFTA than those in SOE and FOE (*P* < 0.05).

**Table 3:** Different characteristics of the BHFTA among the subjects (*n =* 32,121)

Variable	BHFTA [n (%)]				***P*** ^*^
	<25^a^	25–39	40–79 ^b^	≥80^c^	
Sex					< 0.001
Female	5957 (70.41)	2160 (25.53)	332 (3.92)	11 (0.13)	
Male	13,995 (59.15)	6777 (28.64)	2809 (11.87)	80 (0.34)	
Age (years)					< 0.001
< 25	1443 (75.63)	428 (22.43)	37 (1.94)	0 (0.00)	
25–29	3044 (72.63)	1001 (23.88)	145 (3.46)	1 (0.02)	
30–34	2691 (69.21)	991 (25.49)	204 (5.25)	2 (0.05)	
35–39	2824 (61.94)	1292 (28.34)	432 (9.48)	11 (0.24)	
40–44	5257 (59.28)	2615 (29.49)	966 (10.89)	30 (0.34)	
≥ 45	4693 (53.90)	2610 (29.98)	1357 (15.59)	47 (0.54)	
NED (years)					< 0.001
0–4	8937 (66.15)	3564 (26.38)	990 (7.33)	20 (0.15)	
5–9	5217 (64.11)	2148 (26.4)	754 (9.27)	18 (0.22)	
10–14	2177 (58.18)	1079 (28.83)	469 (12.53)	17 (0.45)	
15–19	1156 (57.63)	576 (28.71)	264 (13.16)	10 (0.50)	
20–24	1508 (54.34)	898 (32.36)	362 (13.05)	7 (0.25)	
25–29	699 (48.51)	517 (35.88)	215 (14.92)	10 (0.69)	
≥ 30	258 (50.69)	155 (30.45)	87 (17.09)	9 (1.77)	
Industry type					< 0.001
Manufacturing	14,901 (63.97)	6178 (26.52)	2168 (9.31)	48 (0.21)	
Construction	107 (36.39)	147 (50.00)	39 (13.27)	1 (0.34)	
Mining	1448 (43.34)	1284 (38.43)	582 (17.42)	27 (0.81)	
Others	3496 (67.35)	1328 (25.58)	352 (6.78)	15 (0.29)	
Enterprise type					< 0.001
SOE	5433 (56.24)	3328 (34.45)	880 (9.11)	20 (0.21)	
FOE	1626 (66.48)	659 (26.94)	160 (6.54)	1 (0.04)	
POE	12,893 (64.42)	4950 (24.73)	2101 (10.5)	70 (0.35)	

*Abbreviations*: *BHFTA* binaural high-frequency threshold average, *NED* noise exposure duration, *FOE* foreign-owned enterprise, *SOE* state-owned enterprise, *POE* private-owned enterprise

Footnote. ^a^ BHFTA ≤25 dB, defined as a normal hearing level; ^b^ BHFTA ≥40 dB, defined as the cut-off point for HFHL; ^c^ BHFTA ≥80 dB, defined as severe HFHL

^*^ *P*-value was analysed by the chi-square test, with significance defined at < 0.05

### The proportion of HFHL in the training and validation cohorts

The characteristics of the training cohort and validation cohort associated with the incidence of HFHL are shown in Table 4. The differences in the characteristics of the two cohorts with respect to the incidence of HFHL were all significant (chi-square test, all *P* < 0.01).

**Table 4:** Characteristics of the HFHL workers in the training cohort and validation cohort

Variable	Training cohort (***n*** = 25,732)		***P***^***#***^	Validation cohort (***n*** = 6389)		***P***^***#***^
	n	Positive cases (%)		n	Positive cases (%)	
Sex			< 0.001			< 0.001
Male	87.77	2004 (90.11)		1632	45 (2.76)	
Female	12.23	220 (9.89)		4757	502 (10.55)	
Age (years)			< 0.001			< 0.001
< 25	1525	23 (1.51)		383	5 (1.31)	
25–29	3369	96 (2.85)		822	21 (2.56)	
30–34	3120	129 (4.13)		768	28 (3.65)	
35–39	3675	296 (8.05)		884	69 (7.81)	
40–44	7085	664 (9.37)		1783	181 (10.15)	
≥ 45	6958	1016 (28.99)		1749	243 (30.00)	
NED (years)			< 0.001			< 0.001
0–4	10,840	708 (6.53)		2671	165 (6.18)	
5–9	6521	549 (8.42)		1616	132 (8.17)	
10–14	2967	324 (10.92)		775	76 (9.81)	
15–19	1625	175 (10.77)		381	57 (14.96)	
20–24	2227	246 (11.05)		548	66 (12.04)	
25–29	1152	156 (13.54)		289	33 (11.42)	
≥ 30	400	66 (16.50)		109	18 (16.51)	
Industry type			< 0.001			< 0.001
Manufacturing	18,673	1486 (7.96)		4622	352 (7.62)	
Construction	232	271 (1.64)		62	7 (11.29)	
Mining	2670	472 (17.68)		671	113 (16.84)	
Others	4157	239 (5.75)		1034	75 (7.25)	
Enterprise type			< 0.001			< 0.001
SOE	7762	524 (6.75)		1899	130 (6.85)	
FOE	1978	109 (5.51)		468	32 (6.84)	
POE	15,992	1591 (9.95)		4022	385 (9.57)	

*Abbreviations*: *HFHL* high-frequency hearing loss, defined by a BHFTA ≥40 dB, *BHFTA* binaural high-frequency threshold average, *NED* noise exposure duration, *FOE* foreign-owned enterprise, *SOE* state-owned enterprise, *POE* private-owned enterprise

Footnote. ^#^ *P*-value was assessed by the chi-square test, with significance at < 0.05

### Risk model and HFHL nomogram

**Fig. 3:**
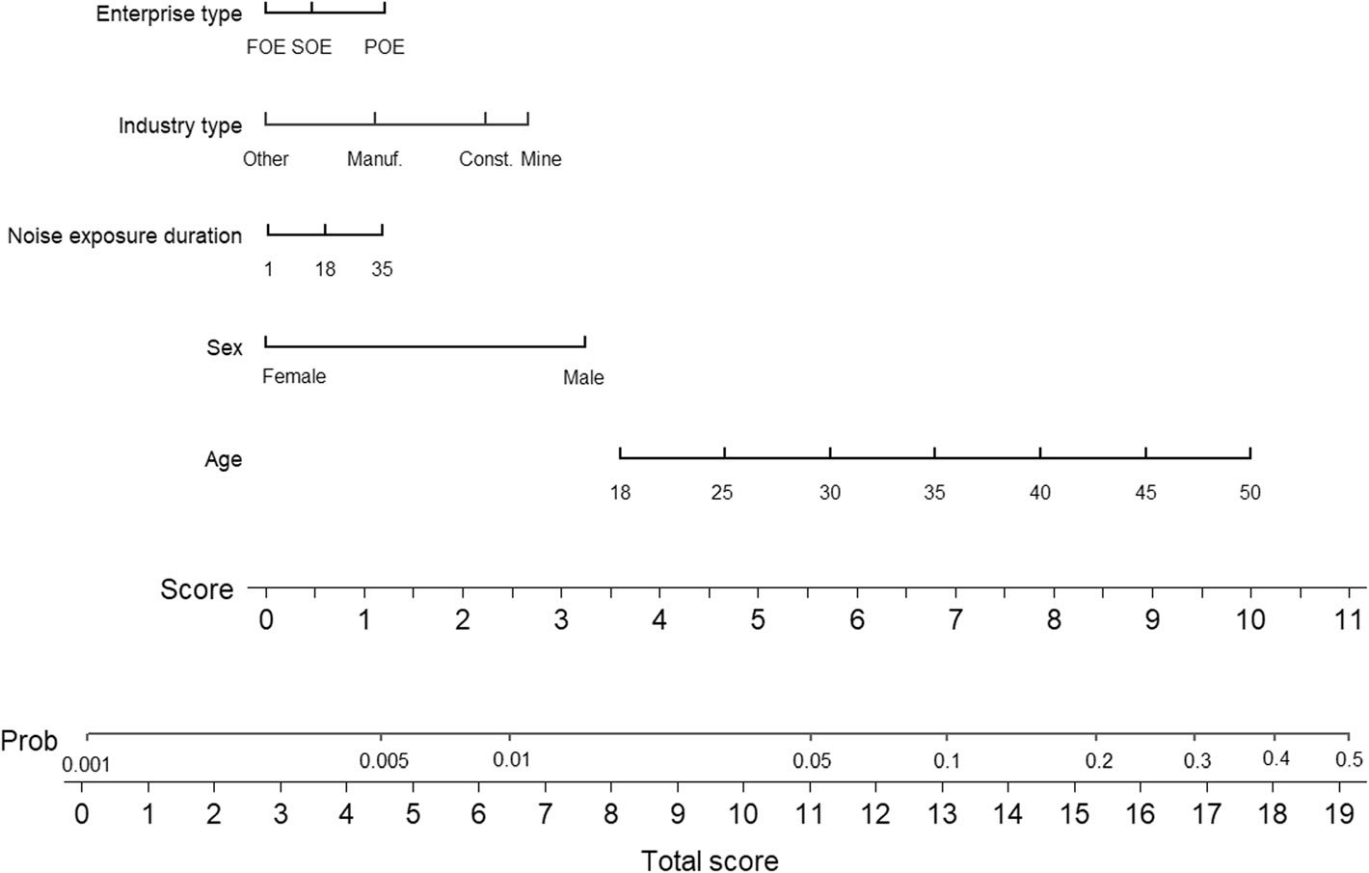
Nomogram for predicting the probability of HFHL in noise-exposed workers. *Abbreviations.* FOE = foreign-owned enterprise, SOE = state-owned enterprise, POE = private-owned enterprise. *Footnote.* The sixth row (points) indicates the points that are assigned to each variable’s measurement from rows 1–5, which are the variables that are included in the risk model. The assigned points for all variables are then summed, and the total value is shown as the total score. Once the total score is located, draw a vertical line down to the bottom line to obtain the predicted probability for HFHL

Five variables were included in the final risk model (Table 5). Growth of age, (OR = 1.09, 95% CI: 1.083–1.104), male sex (OR = 3.25, 95% CI: 2.855–3.702), increase in NED (OR = 1.15, 95% CI: 1.093–1.201), and working in manufacturing (OR = 1.50, 95% CI: 1.314–1.713), construction (OR = 2.29, 95% CI: 1.531–3.421), mining (OR = 2.63, 95% CI: 2.238–3.081), or for a POE (OR = 1.33, 95% CI: 1.202–1.476) were associated with an increased risk of HFHL (all *P* < 0.05). Based on the final risk model, the nomogram was established, which included the five identified variables, to identify the risk probability of HFHL (Fig. 3).

**Table 5:** The risk model for HFHL in the training cohort

Predictor	OR	SE	Z	P*	95% CI (Lower-Upper)
Age	1.09	0.01	18.28	< 0.001	1.083–1.104
Sex					
(Ref: Female)					
Male	3.25	0.22	17.79	< 0.001	2.855–3.702
NED	1.15	0.03	5.64	< 0.001	1.093–1.201
Industry type					
(Ref: Others)					
Manufacturing	1.50	0.10	6.00	< 0.001	1.314–1.713
Construction	2.29	0.47	4.04	< 0.001	1.531–3.421
Mining	2.63	0.21	11.84	< 0.001	2.238–3.081
Enterprise type					
(Ref: SOE)					
FOE	0.88	0.09	−1.20	0.230	0.715–1.084
POE	1.33	0.07	5.47	< 0.001	1.202–1.476

*Abbreviations*: *HFHL* high-frequency hearing loss, *OR* odds ratio, *SE* standard error, *NED* noise exposure duration, *FOE* foreign-owned enterprise, *SOE* state-owned enterprise, *POE* private-owned enterprise

Footnote. ^*^P-values were analysed by binary logistic regression, with significance at < 0.05. In the logistic regression model, “0” was defined as a BHFTA < 40 dB, and “1” was defined as a BHFTA ≥40 dB

**Fig. 4:**
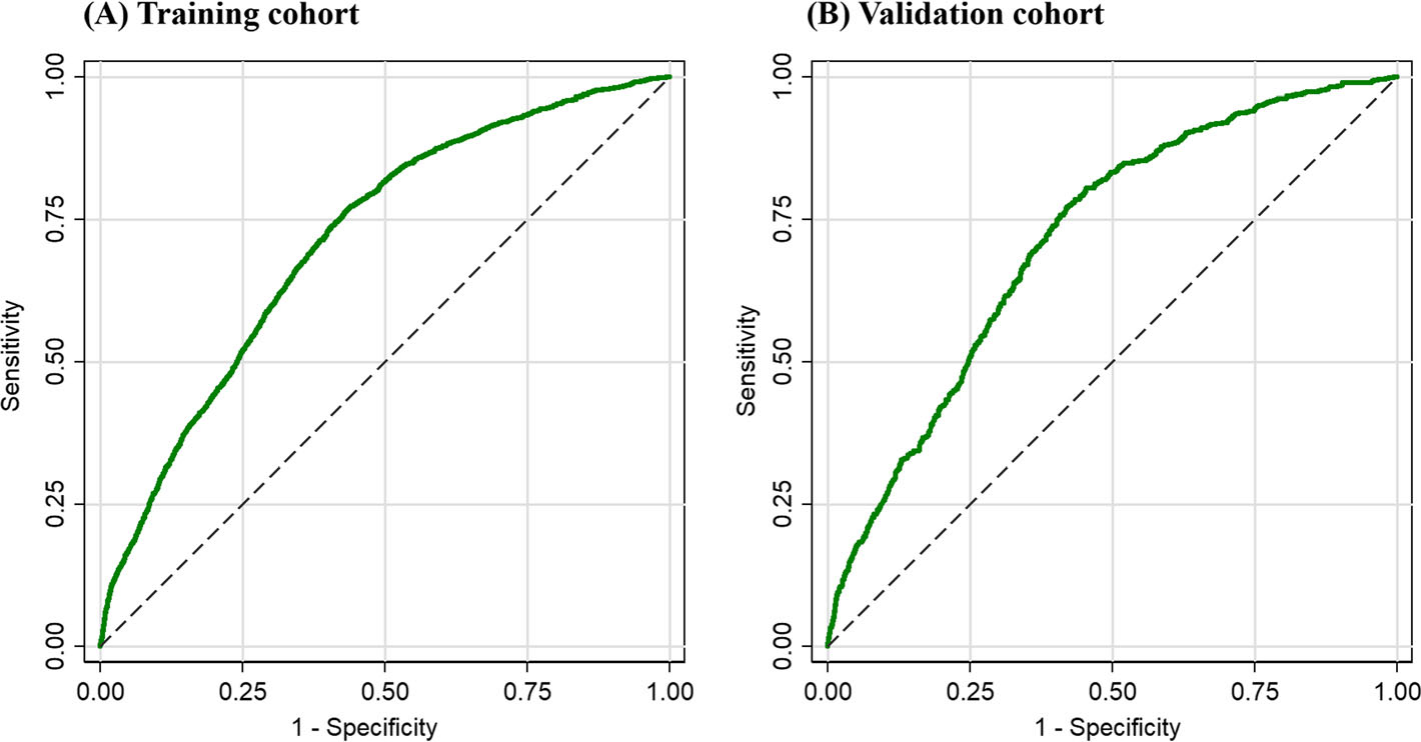
Receiver operating characteristic (ROC) curve showing the performance of the risk model in identifying workers with HFHL in the training cohort (AUC = 0.713) and validation cohort (AUC = 0.714)

The AUC of the ROC curve in the two cohorts is shown in Fig. 4. In the training cohort, the final risk model had good discrimination, as demonstrated by an AUC of the ROC curve of 0.713 (95% CI: 0.704–0.722). In the validation cohort, the model continued to have the excellent discriminatory ability (AUC = 0.714, 95% CI: 0.695–0.733).

**Fig. 5:**
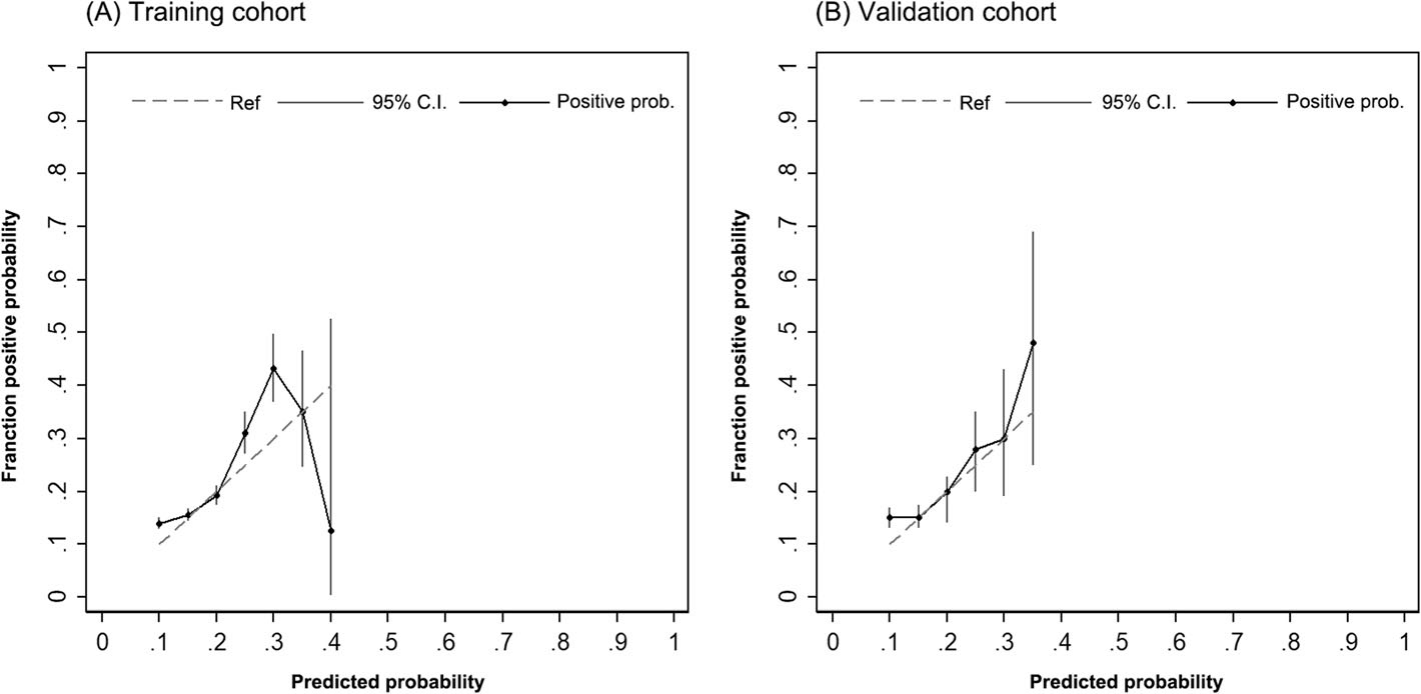
Calibration curve for predicted versus observed risk of HFHL in the training and validation cohorts. The risk model estimated probability is plotted on the X-axis, and the fraction corresponding to the positive probability is plotted on the Y-axis

Figure 5 demonstrates the calibration curves of the risk model in the training cohort and validation cohort. The calibration plots of the models are shown for the two cohorts, which each have a certain degree of deviation. For the validation cohort, the model showed good predictions throughout the range of predicted risks and was accurate through a range of predicted probabilities of 15% to approximately 30% risk of HFHL. The average deviation was 2.0%, with a positive probability less than the predicted probabilities of 30%, while the average deviation was 7.0% with a positive probability greater than 30%.

To ensure the practical applicability of the model, the cut-off point of 9.6% was established based on the maximum Youden’s index and was used to stratify patients into high- and low-risk groups. The sensitivity at the cut-off point was 0.73 and the specificity at the cut-off point was 0.60.

## Discussion

Most research has supported that long-term noise exposure in workers is likely to cause HFHL [35]. In this study, we identified the risk factors for HFHL and also developed a HFHL nomogram for noise-exposed workers.

In our study, 10.06% (3232/32121) of the workers had HFHL. The prevalence of HFHL in Sichuan is higher than that in China. The prevalence is higher than that in Ordos (5.5%), Wuhan (7.3%), Jining (4.6%), and Tianjin (8.3%) [36–39], and lower than that in Zhoushan (22.02%) and Sanmenxia (12.0%) [40, 41]. Occupational hearing loss has historically been difficult to compare because the criteria for HFHL among workers vary from country to country [42].

In our risk model, the results indicated that males (OR = 3.25) experience more effects after exposure to occupational noise than females. The reason may be due to males usually having greater exposure to noise at work than females due to differences in occupational categories and lifetime work history. Several animal and human studies have demonstrated that women may be protected against hearing loss because of oestrogen and its signalling pathways [43, 44]. Age (OR = 1.09) and NED (OR = 1.15) have a negative effect on HFHL, and research has showed that age and NED are important factors for changes in the hearing threshold. Our study strongly supports these results [45–47].

Interestingly, our risk model showed that age and NED had independent effects on HFHL when the BHFTA was adjusted for age. There could be two explanations for this result: first, the study sample is predominantly comprised of male workers, and studies have shown that male workers are generally more vulnerable to occupational hearing loss than female workers [48]. There was a greater proportion of females was used to control for age, which may have accounted for some of the effects. Second, in the risk model, the variable age may represent occupational hearing loss due to other exposures and not the influence of age on HFHL over time, given that the exposure duration is related to age. Exposures to other factors such as ototoxic chemicals in the workplace may not be detected by occupational environmental monitoring. Chemicals alone or combined with noise have recently become a concern as a cause of occupational hearing loss [49]. In the NKODS-Sichuan, noise-exposed workers were usually asked whether they were exposed to toxic chemicals in the workplace, but in general, workers often do not know or remember which chemicals they have encountered. In addition, toxic chemicals may have greater effects with longer exposure durations or in older workers. This may also explain why age and NED had independent effects on HFHL when age was adjusted for in this study. It is difficult to detect toxic chemical exposure in such nationwide screenings for NIHL among noise-exposed workers. However, toxic chemical effects on occupational hearing loss still should be considered in the future [50].

The risk model showed that the mining industry (OR = 2.63) has a strong negative effect on HFHL, with a higher positive rate of HFHL than the other industry types in both the validation cohort and training cohort. Our results suggest that mining workers should have more hearing protective measures and receive more attention from managers. In Poland, according to a report by the Central Statistical Office, the number of workers who exceeded the noise level (85 dB) was approximately 200 thousand, with the highest numbers in industries related to mining, metal and metal product production, textiles and wood production [51]. The mining of minerals necessitates the use of heavy energy-intensive types of machinery and equipment, leading miners to be exposed to high noise levels [52]. Based on the risk model and nomogram, workers who are employed in POE are more likely to experience HFHL than those in other enterprise types. Private enterprises are mostly small-sized enterprises, which are limited by less investment in occupational disease prevention, a lack of occupational health management, and no personal protective equipment. Moreover, the level of noise exposure for those in POE is more serious than those in SOE and FOE, and POE should be regarded as a key enterprise that is in need of prevention and management strategies for HFHL. In addition, the supervision department should pay more attention to the use of hearing protection by noise-exposed workers in POE [53, 54].

We also developed a HFHL nomogram based on the risk model. In the training cohort, the final risk model had good discrimination, as demonstrated by an AUC of the ROC curve of 0.713 (95% CI: 0.704–0.722), and the model continued to have excellent discriminatory ability in the validation cohort (AUC = 0.714, 95% CI: 0.695–0.733). Our nomogram was successfully subjected to independent external validation, which revealed good calibration and better discrimination in the validation cohort than in the training cohort. The results indicate that the risk model and nomogram for HFHL can be an effective tool to assess noise-exposed workers’ risk for HFHL. Moreover, due to its easy-to-use and the visualization features, noise-exposed workers can use the tool on a daily basis. Furthermore, the maximum Youden’s index revealed that noise-exposed workers have a risk probability of HFHL greater than 9.6%. Administrative staff should pay more attention to providing hearing protection for or transferring the position of noise-exposed workers. Regarding further research on the HFHL nomogram, researchers can focus on: (1) comparing other assessment tools with the HFHL nomogram in different occupation-specific populations and (2) the risk probability of HFHL could be considered a new variable in further studies on occupational hearing loss.

This study still has two limitations. (1) Generally, the direct predictor of HL is noise cumulative exposure (NCE), but the NKODS did not collect the NCE levels of the participants. If data on area noise monitoring instead of the NCE levels were included in the model, differences in the results may have been observed. Therefore, we used the NED to represent the level of noise exposure. (2) Exposure to toxic drugs and chemicals was self-reported by the workers in the NKODS. Workers may be unaware of toxic drugs that they have taken or toxic chemicals that may be present in their work environment, which may have affected our results.

## Conclusions

In this study, 10.06% (*n* = 3232) of the workers had HFHL. In the training cohort, we identified that the risk predictors for HFHL were male sex, growth of age, increase in NED, and employment in the manufacturing industry, construction industry, mining industry, or for a POE. Based on the risk model, a nomogram was developed to predict the individualized risk for HFHL among noise-exposed workers. We believe that risk model and nomogram can be used to enhance application-oriented research on HFHL and will support the development of management strategies to prevent occupational hearing loss.

## Supplementary Information

**Additional file 1.**

## Data Availability

The all of the research materials are in the manuscript and request of materials should contact with the corresponding author (e-mail: yajialan501@126.com).

## Abbreviations

WHO: World health organization
NHFPC: National health and family planning commission
OENTD: Occupational ear, nose and throat diseases
HFHL: High-frequency hearing loss
NIHL: Noise-induced hearing loss
GPD: Gross domestic product
FYP: Five-year plan
NKODS: National key occupational disease survey
CDC: Centers for disease control and prevention
PTA: Pure tone audiogram
BHFTA: Binaural high-frequency threshold average
NED: Noise exposure duration
FOE: Foreign-owned enterprise
SOE: State-owned enterprise
POE: Private-owned enterprise
OR: Odds ratio
SE: Standard error
ROC: Receiver operating characteristic
AUC: Area under the curve
