# Supplementary material for: A risk model and nomogram for high-frequency hearing loss in noise-exposed workers
Source: BMC Public Health. 2021 Apr 17;21:747. doi: 10.1186/s12889-021-10730-y (PMC8053268; doi:10.1186/s12889-021-10730-y)
Supplement: Supplementary file 1 — Additional file 1. [file 12889_2021_10730_MOESM1_ESM.docx]

The risk model and nomogram for high-frequency hearing loss in noise-exposed workers

Ruican Sun^1^, Weiwei Shang^2^, Yingqiong Cao^3^, Yajia Lan^1*^

1. Department of Occupational and Environmental Health, West China School of Public Health and West China Fourth Hospital, Sichuan University, Chengdu, Sichuan, China
2. Department of Occupational Health and Radial Control, Sichuan Center for Disease Control and Prevention, Chengdu, Sichuan, China
3. Department of Occupational Disease Prevention and Control, Pidu District Center for Disease Control and Prevention, Chengdu, Sichuan, China

^*^ Correspondence and request of materials should be addressed to Yajia Lan (e-mail: <yajialan501@126.com>).

**Supplementary Material-Table 1.** The selected values of the statistical distribution of hearing threshold levels


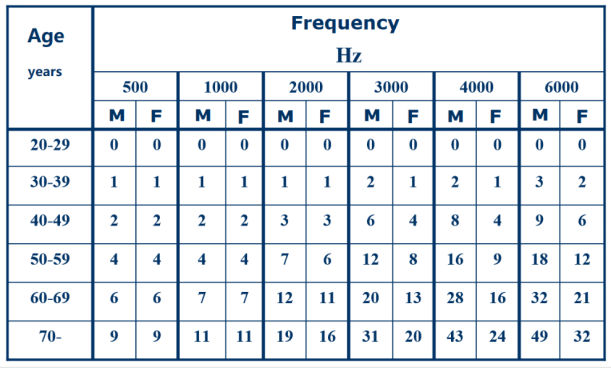


*Footnote.* M=male; F=female; Calculation formula of BHFTA = [the left ear (HL3 kHz + HL4kHz + HL6kHz) +the right ear (HL3kHz + HL4kHz + HL6kHz)]/ 6. The table was derived from the Regulation of Acoustic Statistical Distribution of the Hearing Thresholds as A Function of Age (GB/T 7582-2004)
